# Factors Associated with Pregnancy among Married Adolescents in Nepal: Secondary Analysis of the National Demographic and Health Surveys from 2001 to 2011

**DOI:** 10.3390/ijerph15020229

**Published:** 2018-01-30

**Authors:** Rina Pradhan, Karen Wynter, Jane Fisher

**Affiliations:** Jean Hailes Research Unit, School of Public Health and Preventive Medicine, Monash University, Melbourne 3800, Australia; Karen.Wynter@monash.edu (K.W.); Jane.Fisher@monash.edu (J.F.)

**Keywords:** adolescent pregnancy, risk factors, protective factors, low- and lower-middle income countries

## Abstract

Pregnancy-related morbidity and mortality are much more prevalent among adolescents than adults, particularly in low-income settings. Little is known about risk factors for pregnancy among adolescents in Nepal, but setting-specific evidence is needed to inform interventions. This study aimed to describe the prevalence, and identify factors associated with pregnancy among adolescents in Nepal between 2001 and 2011. Secondary analyses of Nepal Demographic Health Surveys (NDHS) data from 2001, 2006, and 2011 were completed. The outcome was any pregnancy or birth among married adolescents; prevalence was calculated for each survey year. Although the rate of marriage among adolescent women in Nepal decreased significantly from 2001 to 2011, prevalence of pregnancy and birth among married adolescent women in Nepal remains high (average 56%) in Nepal, and increased significantly between 2001 and 2011. Regression analyses of this outcome indicate higher risk was associated with living in the least resourced region, early sexual debut, and older husband. Despite national efforts to reduce pregnancies among married adolescent women in Nepal, prevalence remains high. Integrated, cross-sectoral prevention efforts are required. Poverty reduction and infrastructure improvements may lead to lower rates of adolescent pregnancy.

## 1. Introduction

Pregnancy among adolescent women is associated with high risks to both the mother and her child. Pregnancy-related deaths are twice as common among women aged 15–19 years, than women aged in their twenties [1,2]. Pregnancies during adolescence are also associated with adverse maternal outcomes, including obstructed labour, nutritional anaemia, preterm birth, postpartum infections, unsafe abortion [3], and adverse child outcomes, including infant mortality, foetal growth retardation, and low birth weight [2,3,4,5]. Although births to adolescents occur globally, approximately 95% of these births occur in low-income countries. Due to this burden of morbidity and mortality, adolescent pregnancy is recognized as a public health priority.

Global initiatives, in particular the Declaration of the Millennium Development Goals (MDG) in 2000, have focused on decreasing maternal mortality through improving access to antenatal care and health facilities for women to give birth with support from skilled birth attendants [6]. One of the essential MDG indicators for improving maternal health was a reduction in births to adolescents by 2015. Despite this effort, there is still a high prevalence of adolescent births in low-income countries. About one in five adolescent women have a live birth before the age of 18; these young women are mainly from South Asia and sub-Saharan Africa. This suggests that these initiatives might not be recognizing or addressing the determinants of adolescent pregnancy in these settings.

To date, initiatives in low-income countries have generally been based in the health sector, and focused on improved care for adolescents who are pregnant. Kotelchuck [7] proposes that interventions are more likely to be effective if they take a comprehensive life course approach, in which preventive efforts begin from puberty, continue during secondary schooling, and include improved health care during and after pregnancy, in particular, in the most disadvantaged communities, in order to reduce morbidity and prevent mortality [6].

In Nepal, adolescents comprise 23% of the population, and early pregnancy remains very common [8]. Recognizing the gravity of the problem, the Nepal Government developed Adolescent Sexual Reproductive Health (ASRH) Policy in 2000. Programs initiated under this policy have focused on increasing the availability of and access to “adolescent friendly” sexual and reproductive health services, and health information to reduce the incidence of early marriage and childbearing. Currently, the government has extended “adolescent friendly” health services to 732 health facilities in 49 of 75 districts [9]. The government is implementing, monitoring, and scaling up the “adolescent friendly” reproductive health services and health information program at the national level, in partnership with national and international non-governmental organizations [9].

Adolescent pregnancy is not, however, associated only with lack of access to health services. A systematic review of studies from low and lower-middle income countries [10] found that the risk of adolescent pregnancy was also increased by wider socio-demographic and cultural factors, including limited education, low socioeconomic position, insufficient access to and non-use of contraception, early sexual initiation, and belonging to an ethnic and religious minority group. In order to assess the patterns and prevalence of adolescent pregnancy and to target interventions effectively, each country requires comprehensive, specific data about local risk and protective factors. On this basis, evidence-informed programs can be designed to prevent or reduce pregnancy and motherhood among adolescent women, and to manage consequences when pregnancy occurs.

The aim of this study was to identify factors assessed in Nepal Demographic Health Surveys that are associated with pregnancies or births among married adolescents in Nepal.

## 2. Methods

Secondary analysis of data from the Nepal Demographic Health Surveys (NDHS) in 2001, 2006, and 2011. In this study, the descriptor “adolescent women” is used to refer to married women aged 15–19 years, and “adolescent pregnancy” to pregnancies and births among women aged 15–19 years.

### 2.1. Ethics

For the NDHS surveys, ethics approval was obtained from the Nepal Health Research Council, Kathmandu, Nepal, and ICF Macro Institutional Review Board, Maryland, USA. Informed consent was obtained prior to the structured face-to-face interview [8,11,12]. For this secondary analysis, ethics approval was obtained from the Monash University Human Research Ethics Committee (reference number CF13/910-2013000428).

### 2.2. Data Source

The NDHS data are collected every five years by the Nepal Government Ministry of Health and Population [8]. The standard Demographic Health Survey (DHS) questionnaire, modified for country-specific needs, was used [8,11,12]. One of the purposes of the NDHS is to provide current and reliable information on reproductive health both for the country as a whole, and for urban and rural areas separately.

### 2.3. Procedure

In these surveys, data are collected in a two-stage stratified process: by selecting households first from the ecological divisions of the country, and then, within these, by rural and urban areas [8,11,12]. As most of the population live in rural areas, oversampling of households in urban areas is undertaken to provide estimates with acceptable levels of statistical precision [8,11,12].

Data are collected by trained staff in household-based structured individual interviews with women and men aged 15 to 49 years. All female participants are asked to provide information about their socio-demographic characteristics, marriage, pregnancy history, use of family planning, fertility preferences, antenatal, birth, and postnatal care, child immunization, nutrition, and knowledge on human immunodeficiency virus (HIV) status. For those who are married, data on their husband’s socio-demographic characteristics are also collected.

Data for this study were extracted from the NDHS 2001, 2006, and 2011 surveys (http://www.measuredhs.com). The recruitment rate for each of these surveys was at least 98%. Permission was obtained to use these data for further analysis from MEASURE DHS, which is the monitoring and evaluation body of the DHSs globally. 

### 2.4. Study Variables

The outcome variable for the analysis was any pregnancy or birth among married adolescent women. Women were asked their “age at first birth” (in years) and whether they were “currently pregnant”, with response options “no or unsure” or “yes”. A woman was considered to have had an adolescent pregnancy if her first pregnancy or birth was at any age up to 19 years, or if she was pregnant at the time of the survey, and aged up to 19 years. The analysis was limited to data from married women aged 15–19, because only married women were included in the 2001 survey, even though both married and unmarried women participated in the 2006 and 2011 surveys. The data were weighted to adjust for the stratified cluster sampling design.

Factors which were identified in prior studies as being associated with adolescent pregnancy in lower income settings were selected for this study, providing that the corresponding variables had been collected in all the three waves of the NDHS. Socio-demographic characteristics included women’s highest level of education attained, religion, ethnicity, place of residence (urban/rural), ecological region (mountains, hills or Terai (plain land)), developmental region (Eastern, Central, Western, Mid-Western, Far-Western), and occupation. Household wealth quintile was used as an indicator of a woman’s socioeconomic position. The DHS wealth quintile is a composite indicator which is derived using principal component analysis based on information about housing characteristics and ownership of household durable goods. Households are classified in five categories based on the wealth quintile: poorest, poorer, middle, richer, and richest. Other variables included the woman’s age at first sexual intercourse, and her husband’s age, education, and occupation.

Although lack of access to and non-use of modern contraceptives are established risk factors for adolescent pregnancy [13,14,15,16,17], contraceptive use and intention could not be assessed for this study, because the NDHS only assessed current use of contraceptives. It was not possible to determine whether contraception had been used by the young women before pregnancy, or only after having a child. Exposure and access to various forms of media has also been found to be a risk factor for adolescent pregnancy [18], but could not be assessed in this study, as significant amounts of data were missing in the 2001 dataset.

To ensure groups of sufficient sizes, for meaningful analyses, some variables were recoded. There is considerable diversity in the population in Nepal, with over 125 different castes/ethnic groups and 92 languages [19,20]. Ethnicity was recoded to 5 categories from 60 different response options (2001), 90 different response options (2006), and 10 different response options (2011). These categories were used as they are consistent with the Main Nepal Caste and Ethnic Groups with Regional Divisions and Social Groups used in the 2001 Census in Nepal [21]: Brahaman/Chhetri, Terai/Madhesi, Janajati, Dalits and Other.

About 81% of the Nepali population is Hindu, 9% Buddhist, 4% Muslim, 3% Kirat, 1% Christian, and 0.76% other religions [22]. Based on these data, the five response options offered for religion were recoded into four: Hindu, Buddhist, Muslim, and Other. 

Respondent’s and husband’s highest level of education attained were recoded from four to three categories: “no education”, “primary”, and “secondary or higher education”. Respondent’s occupation at the time of the survey or in the previous 12 months was recoded from seven to four categories: “agricultural work”, “professional work”, “not working”, and “manual work”. In the Nepali context, the term “working” usually refers to income-generating work, and unpaid household or caregiving work is not conceptualised or named as “work”. Therefore, “not working” in this context is assumed to mean not having a paid job. Husband’s occupation was recoded similarly, except “don’t know” was retained as a category; it is not clear whether married adolescent women selected “don’t know” as a response to this question, because they did not know whether their husband was working or because their husband was unemployed.

### 2.5. Statistical Anaysis

The prevalence of adolescent pregnancy was calculated for each survey. Univariate comparisons were conducted to identify possible associations between the relevant socio-demographic and reproductive health factors, and the outcome variable: any adolescent pregnancy. For continuous variables, *t*-tests were conducted, if the variable was normally distributed; if not, non-parametric Mann–Whitney tests were used to test for differences between the two groups (no adolescent pregnancy versus any adolescent pregnancy). Pearson’s Chi-squared analysis was used to test for associations between categorical variables and the outcome variable.

To identify a broad range of explanatory variables that might be associated with pregnancy among married adolescent variables, a less restrictive *p*-value of 0.1 was used as cut-off in univariate analysis [23]. Therefore, all variables with *p* values less than 0.1 in univariate analysis were included in a logistic regression model. In addition, some variables which did not meet these statistical significance criteria, but were expected to be associated with married adolescent pregnancy based on existing studies in similar settings, were also included in the logistic regression model. Odds ratios and 95% confidence intervals were calculated. Statistical significance was set at *p* < 0.05 when considering the multivariate model. IBM SPSS Statistics version 20 (Armonk, NY, USA) was used for the data analysis.

## 3. Results

Data from a total of 2524 married women aged 15–19 from the three NDHS datasets were included in analyses (Table 1). 

### 3.1. Characteristics of Married Adolescent Women

The socio-demographic characteristics of the participants are shown in Table 2. In all three surveys, most of the married adolescent women were living in rural areas, and more than half in the Terai ecological zone. A higher proportion lived in the Central region compared with all other regions in all three surveys. In 2006 and 2011, more than half of married adolescent women were educated at least to primary school level. In all three surveys, most of them followed the Hindu religion, and a higher proportion belonged to the Janajati ethnic group compared with other ethnic groups. A smaller proportion of married adolescents were classified as belonging to households in the “richest” wealth quintile, compared to all other wealth quintiles.

In all three surveys, more married adolescent women worked in the agricultural sector or were “not working” than were engaged in non-agricultural income-generating work. The mean age at first sexual intercourse among married adolescent women was almost 16 years. The mean age of the husbands of these married adolescents was 22 years. Amongst the husbands, a higher proportion had secondary or higher education than primary, or no formal education in all three surveys, and in 2001 and 2006, a higher proportion of husbands were involved in agricultural work than in other occupations.

### 3.2. Changes in the Socio-Demographic Characteristics of Married Adolescent Women and Their Husbands across Time

There was a significant decrease in the proportion of married adolescent women living in rural areas from 2001 to 2006, and then a significant increase to 2011 (Table 2). A significantly higher proportion of women lived in the Far-Western developmental region, and a significantly lower proportion in the Central developmental region in 2006, compared to 2001 and 2011. There was a significant decrease in the proportion of married adolescent women who had no formal education over the years from 2001 to 2006, and from 2006 to 2011. More than half of married adolescent women were educated to secondary or higher levels in 2011, which reflected a significant increase from 2001. There was also a significant increase in the proportion of husbands with secondary or higher education from 2001 to 2006, and again from 2006 to 2011.

There was a significant decrease in the proportion of married adolescents in the poorest wealth quintile from 2001 to 2006, and a significant increase in the proportion of married adolescents in the middle wealth quintile from 2001 to 2006. There was also a significant decrease in the proportion of married adolescent women reporting their occupation as agricultural work from 2006 to 2011, and an increase in the proportion reporting their occupation as manual work and “not working” from 2001 to 2011. There was a significant decrease in the proportion of married adolescents’ husbands working in agricultural jobs from 2001 to 2006, and from 2006 to 2011; a significant increase in proportion of husbands in manual work from 2001 to 2011; and a significant increase in proportion of husbands reported as being in professional work from 2001 to 2006, and again from 2006 to 2011. These findings reflect changes which occurred in standards of living in married adolescent women and their husbands over the decade 2001–2011.

Married adolescent women’s mean age at first intercourse, and husbands’ age, appeared to be stable over the decade. There was no change in the proportion of married adolescent women within ecological zones and religious groups across the three surveys.

### 3.3. Prevalence of Pregnancy or Birth among Married Adolescent Women

The prevalence of pregnancy among married adolescent women was calculated for each survey year, and found to be 53% in 2001, 57% in 2006, and 58% in 2011. Although the proportion of adolescent women who were married decreased significantly from 2001 to 2011 (Table 1), in all three surveys, more than half of the married adolescent women in the sample had a child or were pregnant. The prevalence of adolescent pregnancy or birth among married adolescents increased significantly from 2001 to 2006, but from 2006 to 2011, there was little change.

The following variables were found to be significantly associated with adolescent pregnancy in at least one of the three survey years: place of residence (urban or rural), ecological zone, developmental region, ethnicity, religion, occupation, wealth quintile, participant’s age at first intercourse, and husband’s age. Respondent’s and husband’s highest level of education and husband’s occupation were not significantly associated with adolescent pregnancy in any of the survey years. However, respondent’s and husband’s highest level of education were included in the multivariate model, based on evidence from other studies in similar settings.

Place of residence (urban or rural), ecological zone, and developmental region were highly associated with each other. Developmental region was chosen for inclusion in the multivariate model as an indicator of place of residence, as it was most consistently associated with the outcome variable in all three surveys. There was also a strong association between ethnicity and religion, which occurred because “Muslim” describes an ethnic group (included in the “Other” ethnicity category), but also describes a specific religion. Almost all respondents indicated that they were Hindu; the other three response categories for religion included only a few respondents. The respondents were more broadly distributed in terms of ethnicity; thus, this variable was selected for inclusion in the multivariate model.

The final logistic regression model is shown in Table 3. Year of survey was included, because of the significant changes in distribution of demographic characteristics, as shown in Table 2.

Only four variables were significantly and independently associated with adolescent pregnancy when other factors were controlled. Higher risk of adolescent pregnancy was associated with living in the Eastern developmental region compared to the Central region. Those living in the Eastern region were 1.6 times more likely to experience adolescent pregnancy or birth as those living in the Central region. Women who experienced sexual intercourse at an older age were significantly less likely to have experienced an adolescent pregnancy. Women who had an older husband were at increased odds of adolescent pregnancy. Pregnancy rates among married adolescents were significantly higher in 2006 and 2011, than in 2001.

## 4. Discussion

The most striking finding in this study is that the prevalence of pregnancy and childbirth among married adolescent women was significantly higher in the two most recent surveys, than the earliest survey years. Nepal experienced economic deterioration with political instability during the “Maoist insurgency”, which lasted from 1996 to 2006. Much of the country’s infrastructure was destroyed, and the country’s reconstruction and recovery efforts are ongoing, however, these are not distributed evenly, and are incomplete. Vulnerable groups, including adolescents, experience the impacts of resource constraints, but have little power or autonomy to influence or improve their socioeconomic situations. Nevertheless, the Nepali government has implemented policies and programs, which have been supplemented by initiatives from the non-government sector and international donors, to improve access to adolescent friendly health services [9], to increase girls’ access to education, and to improve compliance with laws about the minimum legal age for marriage of 18 years. The impact of these efforts could be seen in the steady decline in adolescent marriage rates from 2001 onwards (Table 1). However, the mean prevalence of 56% indicates that pregnancy among married adolescents in Nepal remains common, with its associated risks to the life and health of the mother and her child [24,25,26].

The strengths of this study include that it used data from nationally representative samples of households included in the Nepal Demographic Health Surveys, which provide specific information for defined health indicators. Sampling, recruitment, and data collection procedures were similar for all three surveys. Selection bias was minimised by a rigorous sampling strategy. Data from all three surveys were collected in structured individual interviews administered in local languages, which enable people with low literacy, or who are unfamiliar with self-report questionnaires, to participate. The only variation among the surveys was that in 2001, data were collected only from married women, whereas both married and unmarried women were included in the 2006 and 2011 surveys. Overall, however, the data are regarded as high-quality indicators of the socio-demographic characteristics and self-reported health status of the population of Nepal.

A limitation of using NDHS data was that inclusion in analyses of two potentially important explanatory factors (use of contraception and exposure to the media) was not possible, because of the way in which the questions had been asked. Despite these, the data provide valuable evidence about the prevalence and factors associated with pregnancy and birth among married adolescent women in Nepal.

A ten-year time frame is considered quite short to identify substantial changes in national health indicators, so testing for significant interactions between the year of survey and socio-demographic characteristics might not be an accurate indicator of change. However, year of survey was included as a predictor in the logistic regression model, and made a significant, independent contribution when controlling for other variables.

The World Health Organization’s Social Determinants of Health Framework (SDH) Viner, et al. [27] emphasizes the need to understand personal, family, community, and structural or national factors, in order to address health inequalities. The risk factors for adolescent pregnancy identified in this study fit the multilevel, SDH Framework [27]. Living in the least developed region where there is generally low access to basic needs, including education, transport, health facilities, and income-generating work, and where women experience restrictive gender-based roles and responsibilities, and minority ethnic groups can be marginalized, was associated with the highest rates of adolescent pregnancy, and indicates that the major risk is structural. This is consistent with Choe, et al. [28] finding a decade ago that adolescent pregnancy and birth were most common in the least developed rural areas than in the urban areas of Nepal. Similarly, adolescent women living in rural areas were at higher risk of having a baby than those in urban areas in Ethiopia and Nicaragua [18,29].

Even though univariate analyses revealed individual risk factors associated with pregnancy among adolescents; when other factors were adjusted for in multivariable analyses, these were no longer statistically significant. Factors such as the socioeconomic status and education level of individuals were outweighed by the overall development status of the region. Nevertheless, the associations found here and the evidence from other resource-constrained countries, that lack of or low education and low socioeconomic position increase risk for adolescent pregnancy [10,13,14,18,29], suggest that these remain relevant.

The risk associated with younger age at first sexual intercourse identified in this study can be interpreted as operating at both individual and family/community levels of the SDH Framework [27]. Generally, in Nepal, it is socially unacceptable to be sexually active before marriage. Despite laws which specify the minimum legal age for marriage as 18 with parental consent, and 20 without parental consent, early marriage is widely accepted in Nepali society [30]. It leads to early sexual debut, and, because of lack of knowledge about and access to contraception, increases the likelihood of early pregnancy. This finding is consistent with evidence from Kenya, which documented the association between early sexual debut and adolescent childbearing [16]. Choe, et al. [28] also concluded from a survey in 2000 that early marriage and sexual debut were associated with higher risk of adolescent pregnancy in Nepal. Adhikari, et al. [31] concluded that young age of marriage is one of the important risk factors for unintended pregnancy in Nepal.

Age disparities, in which the husband is older than the young woman (in these data, around seven years), also increased risk of pregnancy. Greater age differences between husbands are commonly associated with greater inequalities in power, and this may mean women lack autonomy to implement their own preferences regarding number and timing of children. Oshiro, et al. [32] reported an association between early marriage and intimate partner violence in Nepal, but data about gender-based violence were only collected in the NDHS 2011. It is likely, however, that when young women are married (probably by family arrangement) to older men, they are less able to make decisions about their reproductive lives.

## 5. Conclusions

The findings confirm that the prevalence of pregnancy and birth among married adolescent women remains high in Nepal. When other factors were controlled for, the rate of pregnancy among married adolescents increased from 2001 to 2011. The high prevalence of adolescent pregnancy suggests that while increasing access to adolescent friendly reproductive health services might have had benefits for the health of women once pregnant, it has done little to reduce adolescent pregnancy. The major original finding of this study is that it is structural, rather than individual or community level factors, that are most strongly associated with the outcome. This suggests that integrated, cross-sectoral prevention efforts are required. The problem remains most prevalent in the least resourced region, which indicates that poverty reduction, increased access to education and income-generating work, and improved infrastructure, might lead to lower rates of adolescent pregnancy. However, as specified in the Sustainable Development Goals, gender equality and gender empowerment are essential to ensuring that these strategies are designed and implemented in ways that benefit girls and women. These data indicate that in addition to overall structural changes, it will be essential to continue to address the rights of girls to equality of participation in secondary and post-secondary education, with pathways to income-generating work. Education is required for families about the benefits to health, life-expectancy, and economic productivity of enabling young women to delay marriage and child-bearing beyond adolescence. Access to education about fertility and reproductive health for young people and their families, and to contraception for young women and men, remain essential. In addition to the evidence generated by the NDHS surveys, there is a need for evidence from young women’s and other stakeholders’ perspectives on the cultural and social factors that maintain early marriage and adolescent pregnancy, to inform and increase the effectiveness of national and local strategies.

## Figures and Tables

**Table 1:** Number of married adolescent women from National Demographic and Household Survey; data from 2001, 2006 and 2011.

Participant Numbers	Year of the Survey			Total
	2001	2006	2011	
Total women participants	8726	10,793	12,674	32,193
Total adolescent women participants	2335	2437	2790	7562
Total married women adolescent women participants	940 (40%)	787 (32%)	797 (29%)	2524

**Table 2:** Socio-demographic characteristics of married adolescent women and their husbands for each study year.

Socio-Demographic Characteristics of Married Adolescent Women	Year of the Survey					
	2001		2006		2011	
	%	N	%	N	%	N
Residential location						
Urban	5.5 ^a^	52	10.9 ^b^	86	7.5 ^a^	60
Rural	94.5 ^a^	889	89.1 ^b^	701	92.5 ^a^	738
Ecological zone						
Mountain	6.7 ^a^	63	9.1 ^a^	72	7.4 ^a^	59
Hill	38.5 ^a^	362	35.8 ^a^	282	35.4 ^a^	282
Terai (plain land)	54.8 ^a^	516	55 ^a^	433	57.2 ^a^	456
Developmental region						
Central	34.2 ^a^	322	29.4 ^b^	231	34.1 ^a^	272
Eastern	21 ^a^	198	20.9 ^a^	164	21.9 ^a^	175
Western	18.8 ^a^	177	20.1 ^a^	158	19.4 ^a^	155
Mid-Western	15.6 ^a^	147	15 ^a^	118	14.9 ^a^	119
Far-Western	10.3 ^a^	97	14.5 ^b^	114	9.6 ^a^	77
Education						
No education	52.2 ^a^	491	37 ^b^	291	23.1 ^c^	184
Primary	26 ^a^	245	30.2 ^a^	238	26.2 ^a^	209
Secondary or Higher	21.7 ^a^	204	32.8 ^b^	258	50.7 ^c^	404
Religion						
Hindu	86.8 ^a^	817	86.4 ^a^	680	84.7 ^a^	676
Buddhist	6 ^a^	56	6.4 ^a^	50	7.3 ^a^	58
Muslim	5.4 ^a^	51	6.4 ^a^	50	5.9 ^a^	47
Other	1.8 ^a,b^	17	0.9 ^b^	7	2.1 ^a^	17
Ethnicity						
Brahaman/Chhetri	25.9 ^a^	244	25.6 ^a^	202	23.5 ^a^	187
* Terai/Madhesi Castes	19.7 ^a^	185	14.1 ^b^	111	12.7 ^b^	101
Dalit	20.5 ^a^	193	18.4 ^a^	145	25.3 ^b^	202
Janajati	28.2 ^a^	265	32.2 ^a^	254	32.1 ^a^	256
Other	5.7 ^a^	54	9.6 ^b^	76	6.4 ^a^	51
Occupation						
Professional work	2.3 ^a^	22	1.9 ^a^	15	1.3 ^b^	42
Not working	28.2 ^a^	265	27.7 ^a^	218	36.7 ^b^	293
Agricultural work	67.7 ^a^	636	67.2 ^a^	529	54.2 ^b^	433
Manual work	1.8 ^a^	17	3.2 ^a,b^	25	3.8 ^b^	30
Wealth Quintile						
Poorest	23.6 ^a^	222	19.3 ^b^	152	20.2 ^a,b^	161
Poor	21.3 ^a^	200	23 ^a^	181	23.9 ^a^	191
Middle	22.5 ^a^	212	26.6 ^a,b^	209	29.3 ^b^	234
Richer	21.5 ^a^	202	19.4 ^a^	153	18.4 ^a^	147
Richest	11.2 ^a^	105	11.7 ^a^	92	8.1 ^b^	65
Respondent’s age at first intercourse (mean (SD))	15.67 ^a^	1.44	15.80 ^b^	1.6	15.82 ^b^	2.27
**Married adolescent women’s husbands**						
Husband’s age (mean (SD))	22.00 ^a^	4.05	21.91 ^a^	3.44	22.71 ^b^	4.07
Husbands Education						
No education	24.3 ^a^	228	14.7 ^b^	116	15.4 ^b^	123
Primary	28.0 ^a^	263	30.1 ^a^	237	23.8 ^b^	190
Secondary or Higher	47.8 ^a^	449	55.1 ^b^	434	60.8 ^c^	485
Husband’s Occupation						
Don’t know	3.3 ^a^	29	3.1 ^b^	24	3.2 ^a^	25
Professional work	24.7 ^a^	220	31.4 ^b^	246	38.0 ^c^	294
Agricultural work	43.4 ^a^	386	35.8 ^b^	281	24.8 ^c^	192
Manual work	28.7 ^a^	255	29.7 ^a,b^	233	33.9 ^b^	262

* The term “Terai” is used to describe both an ecological region and an ethnic group. An ethnic group originally from the Terai region is also classified as the Terai/Madhesi group. “^a^”,“^b^”,“^c^” Within each response category, superscript letters denote significant differences between data collection years.

**Table 3:** Logistic regression of factors associated with pregnancy among married adolescent women.

Variables	Proportion of Married Adolescents Who Reported Pregnancy or Birth (%)	Adjusted OR	95% Confidence Interval		*p*-Value
			Lower	Upper	
Developmental Region					
Central (ref)	53.9				
Eastern	64.7	1.59	1.25	2.02	<0.001
Western	53.6	1.04	0.81	1.33	0.78
Mid-Western	55.6	1.28	0.96	1.71	0.09
Far-Western	49.3	1.04	0.76	1.42	0.82
Education					
Secondary or Higher (ref)	54.8				
Primary	57.7	1.09	0.86	1.38	0.49
No education	55.5	1.05	0.81	1.35	0.71
Ethnicity					
Brahaman/Chhetri (ref)	53.6				
Terai/Madhesi Castes	50.5	1.00	0.73	1.37	0.99
Dalit	57.9	1.24	0.94	1.62	0.12
Janajati	59.0	1.49	1.16	1.90	0.001
Other (minority)	60.3	1.29	0.85	1.95	0.24
Occupation					
Professional work (ref)	61.5				
Not working	57.8	0.87	0.51	1.48	0.61
Agriculture work	53.8	0.78	0.47	1.31	0.35
Manual work	72.2	1.69	0.79	3.62	0.17
Wealth Quintile					
Richest (ref)	61.1	0.98	0.55	1.71	0.95
Richer	59.1	0.94	0.68	1.32	0.74
Middle	54.6	0.79	0.56	1.10	0.16
Poor	53	0.80	0.57	1.14	0.22
Poorest	54.7	0.84	0.58	1.21	0.35
Respondent’s age of first intercourse (mean, SD)					
Adolescent pregnancy	15.52 (1.41)	0.68	0.64	0.73	<0.001
No pregnancy	16.06 (2.15)				
Husband’s age (mean, SD)					
Adolescent pregnancy	22.66 (3.76)	1.10	1.08	1.13	<0.001
No pregnancy	21.39 (3.73)				
Husband’s Education					
Secondary or Higher (ref)	55.2				
Primary	57	1.01	0.81	1.25	0.95
No education	55.9	0.87	0.67	1.13	0.29
Year of survey					
Year 2001 (ref)	53.2				
Year 2006	57.3	1.24	1.01	1.53	0.041
Year 2011	57.6	1.28	1.028	1.60	0.028

Ref: Reference category.

## Abbreviations

MDG	Millennium Development Goals
ASRH	Adolescent Sexual Reproductive Health
NDHS	Nepal Demographic Health Surveys
DHS	Demographic Health Survey
HIV	Human Immunodeficiency Virus
SDH	Social Determinants of Health Framework
